# Comprehensive health literacy in Japan is lower than in Europe: a validated Japanese-language assessment of health literacy

**DOI:** 10.1186/s12889-015-1835-x

**Published:** 2015-05-23

**Authors:** Kazuhiro Nakayama, Wakako Osaka, Taisuke Togari, Hirono Ishikawa, Yuki Yonekura, Ai Sekido, Masayoshi Matsumoto

**Affiliations:** College of Nursing, St. Luke’s International University, 10-1 Akashi-cho, Chuo-ku, Tokyo 104-0044 Japan; Faculty of Liberal Arts, The Open University of Japan, Chiba, Japan; Department of Health Communication, School of Public Health, The University of Tokyo, Tokyo, Japan; Department of Hygiene and Preventive Medicine, Iwate Medical University, Iwate, Japan

**Keywords:** Health literacy, Health care, Disease prevention, Health promotion, Health information, Health decision-making

## Abstract

**Background:**

Health literacy, or the ability to access, understand, appraise and apply health information, is central to individuals’ health and well-being. A comprehensive, concept-based measure of most dimensions of health literacy has been developed for the general population in Europe, which enables comparisons within and between countries. This study seeks to validate this tool for use in Japan, and to use a Japanese translation to compare health literacy levels in Japan and Europe.

**Methods:**

A total of 1054 Japanese adults recruited through an Internet research service company, completed a Japanese-language version of the 47-item European Health Literacy Survey Questionnaire (HLS-EU-Q47). The survey was administered via an online questionnaire, and participant demographics were closely matched to those of the most recent Japanese national census. Survey results were compared with those previously reported in an eight-country European study of health literacy.

**Results:**

Internal consistency for the translated questionnaire was valid across multiple metrics. Construct validity was checked using confirmatory factor analyses. The questionnaire correlated well with existing scales measuring health literacy and mental health status. In general, health literacy in the Japanese population was lower than in Europe, with Japanese respondents rating all test items as more difficult than European respondents. The largest difference (51.5 %) was in the number of respondents finding it difficult to know where to get professional help when they are ill.

**Conclusions:**

This study translated a comprehensive health literacy questionnaire into Japanese and confirmed its reliability and validity. Comparative results suggest that Japanese health literacy is lower than that of Europeans. This discrepancy may be partly caused by inefficiency in the Japanese primary health care system. It is also difficult to access reliable and understandable health information in Japan, as there is no comprehensive national online platform. Japanese respondents found it more difficult to judge and apply health information, which suggests that there are difficulties in health decision-making in Japan.

Numerous issues may be linked to lower levels health literacy in Japan, and further studies are needed to improve this by developing individual competencies and building supportive environments.

**Electronic supplementary material:**

The online version of this article (doi:10.1186/s12889-015-1835-x) contains supplementary material, which is available to authorized users.

## Background

As modern societies grow more complex, consumers are increasingly bombarded with health information and, more worryingly, health misinformation [1]. In recent years, health literacy, defined as the ability to access, understand, appraise and apply health information [2], has become an increasingly fundamental component of the pursuit of health and well-being. Problems with achieving health literacy are not unique to any one country. A 2003 US Department of Education literacy assessment found that nearly 90 % of US adults are not proficient in reading, understanding and acting on medical information; according to this survey, one in three patients has “basic” or “below basic” health literacy [3]. In 2006, the Australian Bureau of Statistics found that almost 60 % of adult Australians had low health literacy, meaning that they were not able to make choices effectively or make their voice heard when making health care decisions [4]. In Europe, findings from the recent European Health Literacy Survey (HLS-EU) indicate that 12 % of adults surveyed had inadequate general health literacy, and 35 % had problematic health literacy [5].

The HLS-EU also showed that the percentages of limited health literacy varied considerably between eight European countries, from 29 % in the Netherlands to 62 % in Bulgaria. We were therefore interested in the situation in Japan, with one of the longest life expectancies in the world. However, no similar Japanese data are available, and to date no comparable national health literacy survey has been undertaken. The concept of health literacy has only recently begun to attract attention there, and there was insufficient evidence about it, especially in the general population.

Risky behavior choices, poor health outcomes and increased health care use and costs are common consequences of poor health literacy. Health literacy is important not only for patients in clinical settings, but also for people seeking to promote their own or others’ health, access health services and make appropriate health decisions. In recent years, health literacy has become a core concept of health promotion [6], focusing on the communication between health care providers and people. This dynamic involves both individual skills and supportive environments that make available easily accessible health information, and facilitate interaction between people and their environment [1].

A variety of instruments have been developed to measure health literacy. The test of Functional Health Literacy in Adults (TOFHLA), typically viewed as the standard, measures the ability to comprehend narrative text and to perform computations involving health-related tasks. A short-form version (S-TOFHLA) containing 36 items has been found to yield valid and reliable estimates of overall health literacy. REALM is a validated and widely used instrument that employs word recognition to measure the comprehension domain of health literacy. The Newest Vital Signs (NVS) is reported to be an overall measure of health literacy, consisting primarily of questions requiring participants to read and interpret numerical facts by reading a standard food label. There is also a widely used single-item test intended to identify adults in need of help with printed health material: “How confident are you filling out medical forms by yourself?”

These instruments primarily measure functional health literacy in terms of the acquisition of basic reading and writing skills. Nutbeam proposed a model of health literacy that includes functional health literacy and two additional levels intended to describe a more complex understanding of health care and associated medical issues: interactive health literacy and critical health literacy [7]. Ishikawa et al. applied this three-dimensional model when developing a 14-item health literacy assessment for Japanese adults with diabetes [8]; this same test was later applied to the general population [9]. Another team led by Ishikawa later tested a five-item health literacy assessment for office workers, with this test intended to measure interactive (communicative) health literacy and critical health literacy [10].

The three-dimensional assessments described above have proven meaningful. In 2014, however, a new review article examining 51 health literacy measurements provided evidence that comprehensive validated measurements for diverse populations are needed [11]. The European Health Literacy Survey Questionnaire (HLS-EU-Q47), developed in 2011, can measure personal ability in understanding health-related issues, and also difficult situations that might easily arise without adequate health literacy [12]. The HLS-EU-Q47 is a comprehensive, concept-based measure of most dimensions of health literacy for the general population, which enables comparisons within and between countries [1, 2, 5, 12]. The instrument was derived from a conceptual model that integrates three health-relevant domains (health care, disease prevention, health promotion) and four information-processing competencies (accessing, understanding, appraising, and applying) related to health-relevant decision-making and tasks. Taken together, these domains and competencies create a matrix capable of measuring health literacy with 12 sub-dimensions, operationalized by 47 items. The 47 items were assessed using a four-point self-reported Likert-type scale (very easy, fairly easy, fairly difficult, and very difficult) to measure the perceived difficulty of selected health-relevant tasks.

The HLS-EU-Q47 therefore refers to self-perceived measures of health literacy and reflects interactions between individual competencies and situational complexities or demands. This should to be taken into account when interpreting the survey results and when comparing results between countries. An analysis of a European eight-country survey using the HLS-EU-Q47 showed the assessment was more associated with self-perceived health than NVS for measuring functional health literacy, and measured more direct and comprehensive health literacy [5]. This assessment tool has been translated into ten languages, with additional language translations in process [13]. This study has added to these with a Japanese-language version. More recently, another comprehensive scale covering nine separate dimensions of health literacy, the Health Literacy Questionnaire (HLQ) [14], was developed. For the purposes of this study, and in the interest of comparing between nationwide data in Japan and data already available from eight European countries, we decided to use the HLS-EU-Q47.

The aim of this study was twofold. First, it attempted to translate the HLS-EU-Q47 into Japanese and to execute a Web-based nationwide survey to explore the reliability and validity of this survey in the Japan. Second, it describes the distribution of comprehensive health literacy in Japan compared with the results of the European Health Literacy Survey (HLS-EU).

## Methods

### Participants

Participants were recruited from among those registered with a Japanese Internet research company. The research company had approximately 2.5 million voluntarily registered participants. We wanted to collect data from a minimum of 1000 men and women aged 20 to 69 years. On March 11, 2013, potential respondents (*n* = 6613) were randomly invited via e-mail to participate in a cross-sectional Web-based anonymous health literacy questionnaire.

When determining who to invite to participate, we tried to match participants’ gender, age group and region (we divided the country into eight regions) to the results of the 2010 Japanese census [15]. We accepted emailed responses from potential participants until we reach the targeted number in gender, age group and region. In all, we collected data from 1054 Japanese adults. Participants voluntarily signed an online informed consent form, approved by our institutional review board. The study received prior approval from the Research Ethics Committee of St. Luke’s International University, Japan.

### Measurements

#### Japanese version of the HLS-EU-Q47

To help validate the Japanese translation of the HLS-EU-Q47, we employed several experts in the field of translation: two Japanese translators (to translate the original English version of the survey into Japanese); three native English translators (to translate the new Japanese version of the HLS-EU-Q47 back into English); and a final translator who compared and checked the resulting Japanese- and English-language versions. For further validation, we hired a professional medical interpreter and a non-professional adult who was bilingual in English and Japanese to compare and check the surveys. We checked the translation process and took into consideration the opinions of these last two individuals, and then compiled the final Japanese version questionnaire (see Additional file 1).

The survey’s answer categories were all phrased similarly to “On a scale from very easy to very difficult, how easy would you say it is to understand why you need health screenings?” and were ranked on a four-point Likert-type scale (1 = very difficult, 2 = fairly difficult, 3 = fairly easy, 4 = very easy). In the original orally administered English-language questionnaire, a “don’t know” answer option was not provided, and was only used when stated spontaneously. However, as this survey was a self-administered questionnaire, we included the response “don’t know” to further help assess participants’ level of health literacy. The initial survey also included an item about health promotion efforts in the workplace. In our questionnaire, we added the response “don’t know/not applicable”; this response was coded as a missing value.

Health literacy indices are constructed as a general health literacy index (GEN-HL) comprising all items. This mechanism provides a general overview as well as three sub-indices: health care health literacy index (HC-HL), disease prevention health literacy index (DP-HL) and health promotion health literacy index (HP-HL).

Index scores, as with the original scale, were standardized on a metric between 0 and 50, using the formula: (MEAN-1) × (50/3) [5]. MEAN is the mean of all item responses for each participant. Index scores were computed only for respondents who had rated (validly answered) at least 80 % of the items associated with all indices (*n* = 927).

We defined four levels of health literacy within these indices: 0–25 for “inadequate”, >25–33 for “problematic”, >33–42 for “sufficient” and >42–50 for “excellent”.

### Confirmation of scale validity

We used two scales to confirm scale validity. The First was the Communicative and Critical Health Literacy (CCHL) scale, comprising three items for communicative health literacy (items i–iii) and two items for critical health literacy (items iv–v) [10]. These items asked in Japanese whether participants would be able to (i) collect health-related information from various sources, (ii) extract the information they wanted, (iii) understand and communicate the obtained information, (iv) consider the credibility of the information and (v) make decisions based on the information, specifically in the context of health-related issues. Each item was rated on a five-point scale, ranging from 1, “strongly disagree”, to 5, “strongly agree”. The second was an assessment of eHealth literacy [16] using the Japanese version of eHEALS (J-eHEALS). This survey uses a five-point Likert scale (ranging from 1, strongly disagree, to 5, strongly agree; score range, 8–40) to measure perceived eHealth literacy.

### Demographic and socioeconomic characteristics

The following demographic and socioeconomic characteristics were analyzed:

- Gender (men, women)
- Age groups (20–29, 30–39, 40–49, 50–59, 60–69)
- Highest level of education (junior high school, high school, 2-year college, college/university, graduate school)
- Annual pre-tax household income in millions of yen (<2.5, 2.5–3.5, 3.5–4.5, 4.5–6.0, 6.0–8.5, 8.5–12.5, ≥ 12.5, unknown)
- Self-assessed living conditions (very hard, a little hard, common, a little well, very well) [17]
- Occupation (self-employed, managerial and administrative, professional and technical, other regular staff [routine and manual], part-time, homemaker, student, unemployed)
- Municipality size (very large [population of 500,000≤], large [100,000–499,999], moderate [<100,000], small)

### Health status

Self-rated health (SRH) was measured by participants’ responses to the question, “Recently, how would you describe your state of health?” Five response categories were available: five = good, four = fairly good, three = fair, two = fairly poor and one = poor. We used the same question and response that appeared in a survey administered by the Ministry of Health, Labour and Welfare to be able to compare our findings against national Japanese data [17]. Additionally, we included in our survey questions from the Five-Item Mental Health Inventory (MHI-5); this tool comprises the following five questions: “How much of the time during the past month (i) have you been a very nervous person?, (ii) have you felt calm and peaceful?, (iii) have you felt downhearted and blue?, (iv) have you been a happy person?, and (v) have you felt so down in the dumps that nothing could cheer you up?” [18, 19]. For these questions, we used a five-point Likert scale ranging from one (all the time) to five (never). All scores were totaled, with higher scores indicating better mental health.

### Statistical analysis

#### Reliability and validity

Cronbach’s alphas were calculated to examine internal consistency. For construct validity, confirmatory factor analysis (CFA) was conducted separately for the three domains of health (health care, disease prevention, and health promotion). The number of factors was set to four related to the four information-processing competencies (accessing, understanding, appraising, and applying). In CFA, the Comparative Fit Index (CFI) and the root mean square error of approximation (RMSEA) were used as the model fit indices. A CFI value of .90 or larger is generally considered to indicate acceptable model fit. RMSEA value of less than .05 represents good fit, and a value < .08 is acceptable [20].

Construct validity was also assessed through the calculation of a Pearson or Spearman’s correlation coefficient between four health literacy indices (GEN-HL, HC-HL, DP-HL, HP-HL) and two other scale of health literacy (CCHL, J-eHEALS) and health status (SRH and MHI-5). When CCHL and J-eHEALS were compared with the HLS-EU-Q47, both scales were narrower concepts; CCHL because it did not contain a functional health literacy measurement and J-eHEALS because it was limited to electronic health information. No strong correlations were expected, but moderate correlations from .4 to .6 were thought likely. In the HLS-EU survey, the correlations between GEN-HL and self-assessed health varied from .15 to .33 across the eight countries [5], so similar correlations between health literacy indices and health status (measured by SRH and MHI-5) were expected.

As to GEN-HL, we compared the mean and standard deviation (SD) by demographic and socioeconomic characteristics and a multiple linear regression analysis was used to explore the associations between GEN-HL and demographic and socioeconomic characteristics (gender, age group, education, income, living conditions, occupation and municipality size). To assess GEN-HL’s associations with health status, multiple linear regression analyses were used to explore the associations between health status (SRH, MHI-5) and GEN-HL, together with demographic and socioeconomic characteristics.

### Comparison between Japan and Europe

The European Health Literacy Survey (HLS-EU) was conducted in 2011 across eight European countries (Austria, Bulgaria, Germany, Greece, Ireland, Netherlands, Poland, and Spain); its findings are openly available online [5]. The accompanying report shows the answer distributions of the survey’s 47 health literacy items, displaying the combined answer categories “very difficult” and “fairly difficult”, the mean percentage of respondents perceiving the items as difficult in the total sample of eight countries.

For the purposes of our research, using the published HLS-EU report as a starting point, we compared the rate of difficulty with health literacy items between Japan and Europe. The reason why we combined “very difficult” and “fairly difficult” in our analysis is that “very” and “fairly” had a subtle difference of nuance in the language of each country, making possible differences in responses. The judgment as to whether something was difficult or easy to understand is, however, rarely problematic in responses between languages. We also compared the mean and standard deviation of the four health literacy indices (GEN-HL, HC-HL, DP-HL, HP-HL) and the distribution of proportions of the categorized index.

Data were analyzed using IBM SPSS Statistics and Amos version 23.0.

## Results

### Participant characteristics

The sample distribution in terms of gender, age and income (Table 1) was in accordance with the distribution of these metrics in the Japanese population (not tabulated) [15]. Unemployed, college/university or higher and very large municipality size were slightly overrepresented, and self-employed, junior high school and large municipality size were slightly underrepresented in the studied population. Regarding self-assessed living conditions, “very hard” was slightly underrepresented in our study as compared with a recent national study of Japanese living conditions, while “common” was slightly overrepresented [17].

**Table 1:** Characteristics of study participants and their general health literacy (GEN-HL)

	Total (*n* = 1054)		GEN-HL (*n* = 927)		One-way ANOVA	
Variables	n	%	mean	SD	F	*P*
Gender						
Men	509	48.3	24.4	8.3	12.17	<.001
Women	545	51.7	26.2	8.1		
Age group						
20 − 29	154	14.6	22.8	7.8	13.06	<.001
30 − 39	228	21.6	23.7	7.6		
40 − 49	211	20.0	24.6	7.3		
50 − 59	213	20.2	26.0	8.4		
60 − 69	248	23.5	28.2	8.8		
Age (mean ± SD)	46.1	13.5				
Highest level of education						
Junior high school	25	2.4	25.7	8.1	0.21	.93
High school	432	41.0	25.4	8.6		
2-year college	142	13.5	25.1	7.2		
College/university	417	39.6	25.2	8.3		
Graduate	38	3.6	26.4	7.7		
Annual pre-tax household income (million yen^a^)						
<2.5	146	13.9	24.2	8.2	0.99	.44
2.5 − 3.5	126	12.0	25.1	9.0		
3.5 − 4.5	119	11.3	25.0	8.6		
4.5 − 6.0	183	17.4	26.3	8.0		
6.0 − 8.5	181	17.2	25.2	7.6		
8.5 − 12.5	92	8.7	25.3	7.8		
12.5 or >	44	4.2	25.9	8.6		
Unknown	163	15.5	27.3	8.6		
Self-assessed living conditions						
Very hard	132	12.5	24.0	9.1	4.81	<.001
A little hard	324	30.7	24.6	8.4		
Common	501	47.5	25.9	8.1		
A little well	87	8.3	26.7	7.3		
Very well	10	.9	32.8	6.6		
Occupation						
Self-employed	61	5.8	25.9	9.8	1.98	.056
Managerial and administrative	38	3.6	23.5	7.0		
Professional and technical	142	13.5	26.4	8.2		
Other (routine and manual)	288	27.3	24.4	8.2		
Part-time	123	11.7	25.7	7.5		
Homemaker	217	20.6	26.5	7.7		
Student	26	2.5	23.5	6.9		
Unemployed	159	15.1	24.7	9.2		
Municipality size						
Very large (population of 500,000 or more)	340	32.3	26.1	8.9	2.09	.10
Large (population of 100,000 − 499,999)	388	36.8	25.3	7.6		
Moderate (population of <100,000)	247	23.4	24.9	8.3		
Small	79	7.5	23.6	8.1		

^a^$1 = about 120yen, in May 2015

### Response to questionnaire

Table 2 shows the exact wordings of the 47 health-literacy items asked of participants, along with the empirical answer patterns for the total sample.

**Table 2:** Percentage of respondents giving each response for all health literacy items

	Health domain/Competence	Items	Very easy	Fairly easy	Fairly difficult	Very difficult	Don’t know/Not applicable	Total difficult (“Very difficult” and “Fairly difficult”)		
								Japan	Europe^a^	Differences
Q1	Health care/Accessing	Finding information on symptoms of illnesses that concern you	7.7	41.4	37.8	8.3	4.8	46.1	22.8	23.3
Q2		Finding information on treatments of illnesses that concern you	6.0	36.0	41.3	12.0	4.7	53.3	26.9	26.4
Q3		Finding out what to do in case of a medical emergency	3.5	30.5	42.6	18.3	5.1	60.9	21.8	39.1
Q4		Finding out where to get professional help when you are ill	4.7	27.4	42.9	20.5	4.5	63.4	11.9	51.5
Q5	Health care/Understanding	Understanding what your doctor says to you	8.3	45.5	37.8	6.3	2.1	44.0	15.3	28.7
Q6		Understanding the leaflets that come with your medicine	11.4	46.1	31.8	9.0	1.7	40.8	28.0	12.8
Q7		Understanding what to do in a medical emergency	3.7	28.5	46.3	17.2	4.4	63.5	21.7	41.8
Q8		Understanding your doctor’s or pharmacist’s instructions on how to take a prescribed medicine	18.3	54.5	20.6	5.0	1.6	25.6	6.5	19.1
Q9	Health care/Appraising	Judging how information from your doctor applies to you	7.4	43.1	39.8	6.8	2.8	46.7	18.0	28.7
Q10		Judging the advantages and disadvantages of different treatment options	3.4	21.6	48.4	22.2	4.4	70.6	42.6	28.0
Q11		Judging when you may need to get a second opinion from another doctor	2.9	16.2	45.1	27.9	7.9	73.0	38.6	34.4
Q12		Judging if the information about illness in the media is reliable	2.8	17.6	51.4	21.8	6.5	73.2	49.7	23.5
Q13	Health care/Applying	Using information the doctor gives you to make decisions about your illness	5.4	39.8	40.5	8.8	5.5	49.3	23.1	26.2
Q14		Following the instructions on medication	27.8	54.0	13.6	3.2	1.4	16.8	6.8	10.0
Q15		Calling an ambulance in an emergency	19.9	38.4	26.8	10.1	4.8	36.8	8.8	28.0
Q16		Following instructions from your doctor or pharmacist	25.1	57.6	12.3	3.1	1.8	15.5	5.6	9.9
Q17	Disease Prevention/Accessing	Finding information about how to manage unhealthy behavior such as smoking, low physical activity and excessive drinking	17.2	52.1	22.4	5.9	2.5	28.3	14.7	13.6
Q18		Finding information on how to manage mental health problems such as stress or depression	6.4	36.1	39.1	13.9	4.6	52.9	33.5	19.4
Q19		Finding information about vaccinations and health screenings that you should have	10.5	45.3	32.8	7.3	4.1	40.1	24.0	16.1
Q20		Finding information on how to prevent or manage conditions such as being overweight, high blood pressure or high cholesterol	12.0	49.8	29.2	5.5	3.5	34.7	18.1	16.6
Q21	Disease Prevention/ Understanding	Understanding health warnings about behavior such as smoking, low physical activity and excessive drinking	36.9	45.9	12.3	3.6	1.2	15.9	10.3	5.6
Q22		Understanding why you need vaccinations	22.5	53.1	17.5	4.3	2.7	21.7	16.6	5.1
Q23		Understanding why you need health screenings	24.5	54.6	15.8	3.3	1.8	19.2	10.4	8.8
Q24	Disease Prevention/Appraising	Judging how reliable health warnings are, such as smoking, low physical activity and excessive drinking	22.1	49.0	21.9	3.9	3.1	25.8	14.4	11.4
Q25		Judging when you need to go to a doctor for a check-up	9.4	33.6	43.6	9.6	3.8	53.2	16.3	36.9
Q26		Judging which vaccinations you may need	7.3	30.2	44.9	12.1	5.5	57.0	32.7	24.3
Q27		Judging which health screenings you should have	9.4	34.2	40.5	12.2	3.7	52.8	25.1	27.7
Q28		Judging if the information on health risks in the media is reliable	5.0	25.1	49.1	15.1	5.6	64.2	42.1	22.1
Q29	Disease Prevention/ Applying	Deciding if you should have a flu vaccination	16.9	43.5	27.7	8.2	3.7	35.9	26.2	9.7
Q30		Deciding how you can protect yourself from illness based on advice from family and friends	7.5	38.4	39.1	9.4	5.6	48.5	22.2	26.3
Q31		Deciding how you can protect yourself from illness based on information in the media	6.4	36.5	42.5	9.6	5.0	52.1	36.9	15.2
Q32	Health Promotion/ Accessing	Finding information on healthy activities such as exercise, healthy food and nutrition	12.7	54.1	24.6	5.3	3.3	29.9	14.3	15.6
Q33		Finding out about activities that are good for your mental well-being	14.2	53.9	22.5	4.8	4.6	27.3	22.6	4.7
Q34		Finding information on how your neighborhood could be more health-friendly	7.9	38.3	39.8	8.2	5.9	47.9	40.3	7.6
Q35		Finding out about political changes that may affect health	5.2	25.8	44.6	18.5	5.9	63.1	53.2	9.9
Q36		Finding out about efforts to promote your health at work	7.9	35.7	30.6	7.3	18.5	38.0	34.8	3.2
Q37	Health Promotion/ Understanding	Understanding advice on health from family members or friends	12.0	52.0	25.9	4.6	5.6	30.5	13.0	17.5
Q38		Understanding information on food packaging	11.0	43.8	32.8	9.0	3.3	41.8	36.2	5.6
Q39		Understanding information in the media on how to get healthier	11.7	51.5	28.6	5.0	3.2	33.6	23.3	10.3
Q40		Understanding information on how to keep your mind healthy	6.9	39.4	39.9	9.4	4.4	49.3	26.1	23.2
Q41	Health Promotion/ Appraising	Judging how where you live affects your health and well-being	5.4	24.7	46.9	14.9	8.2	61.8	24.6	37.2
Q42		Judging how your housing conditions help you to stay healthy	6.4	27.4	46.2	12.7	7.3	58.9	19.5	39.4
Q43		Judging which everyday behavior is related to your health	10.3	41.2	36.1	9.5	2.9	45.5	12.6	32.9
Q44	Health Promotion/ Applying	Making decisions to improve your health	10.2	36.8	36.9	13.8	2.3	50.7	21.7	29.0
Q45		Joining a sports club or exercise class if you want to	9.1	29.4	36.8	19.5	5.1	56.4	24.1	32.3
Q46		Influencing your living conditions that affect your health and well-being	6.5	26.9	43.6	19.9	2.9	63.6	25.5	38.1
Q47		Taking part in activities that improve health and well-being in your community	4.8	23.3	44.7	19.9	7.2	64.6	38.9	25.7

^a^8 countries, total *n* = 8102

The percentage distributions show that there is considerable variation in item difficulty, ranging from 2.8 % (Q12 “Judge if the information about illness in the media is reliable”) to 36.9 % (Q21 “Understand health warnings about behavior such as smoking, low physical activity and drinking too much”) for “very easy”, and from 3.1 % (Q16 “Follow instructions from your doctor or pharmacist”) to 27.9 % (Q11 “Judge when you may need to get a second opinion from another doctor”) for “very difficult”.

### Reliability and validity

Internal consistency was excellent for the four health literacy indices examined (GEN-HL, HC-HL, DP-HL and HP-HL, Cronbach’s alpha = .97, .92, .93 and .94) (Table 3). In accordance with the three domains defined, confirmatory factor analysis was conducted. The CFI and RMSEA values were .937 and .075 (health care domain), .943 and .079 (disease prevention domain), and .934 and .078 (health promotion domain), which indicated acceptable fit.

**Table 3:** Reliability and validity of health literacy indices

	Cronbach’s alpha	Pearson’s correlation coefficients			
		CCHL	J-eHEALS	SRH^a^	MHI-5
Health literacy indices					
GEN-HL	.97	.62	.51	.18	.26
HC-HL	.92	.57	.47	.12	.21
DP-HL	.93	.55	.44	.17	.24
HP-HL	.94	.57	.49	.22	.26
CCHL				.17	.16
J-eHEALS				.13	.10^b^

^a^Spearman’s correlation coefficients

All correlation coefficients are significant (*P* < .001 except for ^b^ *P* = .036)

The general health literacy index (GEN-HL) mean score was significantly higher in women than in men, and increased with age groups and better self-assessed living conditions (Table 1). We also conducted a multiple regression analysis with GEN-HL as the dependent variable, and independent variables of demographic and socioeconomic characteristics (gender, age groups, education, income, occupation, self-assessed living conditions, municipality size). Gender (F = 11.42, *P* = .001), age groups (F = 12.41, P < .001) and self-assessed living conditions (F = 2.59, *P* = .036) were significantly associated with GEN-HL.

Bivariate relationships between health literacy indices and CCHL, J-eHEALS, SRH and MHI-5 are shown in Table 3. GEN-HL had the highest correlation with CCHL and J-eHEALS (.61 and .51). J-eHEALS, which was specific to health information on the Internet, had a slightly lower correlation.

GEN-HL and HP-HL were slightly correlated with SRH, with correlations of about .2 (.18 and .22). GEN-HL and HP-HL were also slightly correlated with MHI-5, at .26; this was the highest correlation. CCHL was similarly slightly correlated with SRH, with a value of .17. The correlation of .16 with MHI-5 was less than GEN-HL and HP-HL. J-eHEALS showed the lowest correlations. Although these correlations are low, they are similar to those found in the European study.

We conducted multivariate analyses to confirm that GEN-HL had significant correlations with health status controlling demographic and socioeconomic characteristics. Multiple linear regression was used. We found that GEN-HL significantly predicted health status from both measures, when controlling gender, age group, education, income, occupation, self-assessed living conditions and municipality size). The standardized coefficients (β) of GEN-HL were .13 (when SRH was dependent variable) and .19 (when MHI-5 was the dependent variable) and both were significant (P < .001) (not tabulated). The new health literacy indices had correlations of .5 to .6 with existing health literacy scales, and higher correlations with health status than either of these. These results support the construct validity of the scales.

### Comparison between Japan and Europe

Table 2 shows that all rates of difficulty (“very” + “fairly”) in Japan were higher than in Europe. The mean of the differences was 21.8 %. The largest difference was 51.5 % (Q4 ‘Find out where to get professional help when you are ill’); the second-largest was 41.8 % (Q7 ‘Understand what to do in a medical emergency’); and the third-largest was 39.4 % (Q42 ‘Judge how your housing conditions help you to stay healthy’). In examining 12 sub-dimensions (four competencies in three domains), we observed items with differences of more than 20 % in all four competencies (“Accessing”, “Understanding”, “Appraising” and “Applying”) in the “health care” domain and in two competencies (“Appraising” and “Applying”) in the “disease prevention” and “health promotion” domains.

Table 4 shows the mean and standard deviation (SD) of GEN-HL, which was 33.8 ± 8.0 in Europe, and 25.3 ± 8.2 in Japan. There was a difference of about one SD. The smallest of the three sub-indices was HP-HL in Japan; this had a large difference from Europe. DP-HL and HP-HL had similar differences with GEN-HL. Although the differences as a whole were about one SD, there were few differences in standard deviations between Japan and Europe.

**Table 4:** Comparison of health literacy indices between Japan and Europe

Health literacy indices			Japan	Europe
GEN-HL	Mean		25.3	33.8
	SD		8.2	8.0
	Categorized index (%)	Excellent	4.2	16.5
		Sufficient	10.4	36.0
		Problematic	35.5	35.2
		Inadequate	49.9	12.4
		Limited^a^	85.4	47.6
HC-HL	Mean		25.7	34.7
	SD		8.6	8.3
	Categorized index (%)	Excellent	2.4	19.9
		Sufficient	18.1	39.1
		Problematic	30.5	28.8
		Inadequate	40.9	12.1
		Limited^a^	71.4	40.9
DP-HL	Mean		22.7	34.2
	SD		9.2	8.8
	Categorized index (%)	Excellent	2.0	21.3
		Sufficient	10.2	35.9
		Problematic	27.6	21.9
		Inadequate	60.1	13.7
		Limited^a^	87.7	35.6
HP-HL	Mean		25.5	32.5
	SD		9.2	9.1
	Categorized index (%)	Excellent	2.6	15.6
		Sufficient	18.3	33.5
		Problematic	33.0	30.8
		Inadequate	46.1	20.1
		Limited^a^	79.1	50.9

^a^“Problematic” and “inadequate”

The percentages of “excellent” in the four categorized indices ranged from 2.0 to 4.2 % in Japan and from 15.6 to 21.3 % in Europe, and the proportions in Japan were lower, at about 15 % (Table 4). The percentages of “sufficient” ranged from 10.2 to 18.3 % in Japan and from 33.5 to 39.1 % in Europe, and in Japan were lower, at about 20 %. By contrast, the percentages of “inadequate” ranged from 40.9 to 60.1 % in Japan and from 12.1 to 20.1 % in Europe; for Japan, these were higher, at about 30–40 %. Combined responses of “inadequate” and “problematic” indicated “limited” of GEN-HL were 47.9 % in Europe and 85.4 % in Japan; there was a difference of 37.5 %. In addition, for this dimension there were variations in the results of the eight European countries; there, the proportion varies from 28.7 % in the Netherlands to 62.1 % in Bulgaria (complete data not shown), while the largest difference from Japan was 56.7 %.

## Discussion

In this study, we successfully developed instruments to measure comprehensive health literacy in Japan, and used them to compare levels of health literacy in Japan and Europe.

Internal consistency reliability was supported with Cronbach’s alpha of .97 for GEN-HL, which was the same value as in the HLS-EU Survey. Cronbach’s alphas of HC-HL, DP-HL and HP-HL were slightly higher than .91, .91 and .92 in Europe, respectively. Construct validity was confirmed through confirmatory factor analyses, which supported the factor structure of the three indices (HC-HL, DP-HL and HP-HL).

These instruments had correlations greater than .5 with existing scales measuring health literacy (CCHL and J-eHEALS), and higher correlations than existing scales with the mental health status (MHI-5). This shows that the instruments discussed here were more comprehensive and predictive of health status, and supported construct validity.

We added the response “don’t know/not applicable” to our questionnaire, which was not formally included in the European version, although it was recorded if participants responded in that way. The percentage of “don't know/not applicable” was highest for item Q36 “Find out about efforts to promote your health at work”, at 18.5 %. This was similar to the 17.1 % of respondents who gave this response in the results of HLS-EU. Excluding this item, the average of “don't know/not applicable” responses for the remaining 46 items was 4.2 %. This suggests that including this option had little impact on the responses.

In our study, health literacy increased with age. In the HLS-EU survey [5], however, older groups tended to have lower health literacy in most countries, although in Ireland and Germany, the correlations were not significant, and in the Netherlands, the correlation was significantly positive. Education level was also associated with health literacy in Europe, but we did not find this, and neither did the Japanese Web survey using the eHealth Literacy Scale (J-eHEALS) [16]. This may be because of interactions between age, education, and active Internet use, because the Web survey sample only included active Internet users, who may have had different characteristics from the general population.

In general, the Japanese population is thought to have more difficulties in achieving health literacy than do European populations. This perception is rooted in the fact that in the past there have been fundamental problems in how Japanese citizens are able to access reliable and understandable health information. In Japan, there is no reliable, understandable, neutral and comprehensive website comparable to MedlinePlus (US National Library of Medicine), as Japan has neither a National Institute of Health nor a National Library of Medicine. When Japanese people search the Internet for information about their symptoms or diseases, they often find unreliable websites. There are numerous reliable disease-specific health websites created and managed by specialist physicians and researchers. These websites are valuable, but are not always accessible, understandable or usable by people with low health literacy because of the shortage of Japanese health communication specialists. An online database for searching Japanese medical literature now exists, but unlike PubMed, it is not neither barrier-free nor available free of charge. It is not easy for the general public to find and read abstracts of Japanese research papers with interesting titles or authors. It therefore makes sense that about half of the respondents answered that it was “difficult” to “Find information about symptoms of illnesses that concern you” and “Find information about treatments of illnesses that concern you”.

There is another reason it is difficult to find health information in Japan, which is the inefficiency of the Japanese primary health care system [21]. This may also be one of the reasons for the difference between the 64 % of Japanese respondents who answered that it was “difficult” to “Find out where to get professional help when you are ill” and the 11.9 % of European respondents who did so. The majority of primary care physicians in Japan do not fit the standard definition of this category. There is a scarcity of gatekeepers (i.e., primary care physicians or nurses) who are trained as generalists. In November 2014, the number of Japan Primary Care Association-certified family physicians was only 452 [22] out of approximately 300,000 physicians in Japan. No education system existed to train medical students and graduates as generalists and family physicians until 2004. This shortage of primary care physicians allows patients to self-refer to secondary or tertiary care hospitals, even when their conditions could be treated as well, if not better, in primary care level [21]. Although there was unrestricted access to any doctor, a considerable number of people were unable to make a quick choice of hospital and doctor because there was too little reliable information. In contrast, in the eight European countries that participated in the HLS-EU study, on average, about 30 % of doctors are general medical practitioners (general practitioners and other generalists) [23]. However, not all of the eight European countries have a ‘gate keeper’ system, so this system can only be part of the reason for the difference.

There were differences of about 30 percentage points between Japan and Europe in the information-processing competences of “Appraising” (i.e., Q9 “Judge how information from your doctor applies to you”, Q44 “Make decisions to improve your health”) and “Applying” (i.e., Q13 “Use information the doctor gives you to make decisions about your illness”, Q30 “Decide how you can protect yourself from illness based on advice from family and friends”). This discrepancy suggests that there may be difficulties in “judging” or “decision-making” in health in Japan. The background of this issue may lay in the fact that very few studies have tried to examine how to provide information about decision-making in health and then support the decision.

Although this study showed that health literacy was lower in Japan than in Europe, Japanese people continue to enjoy one of the longest life expectancies in the world. Many factors contribute to this, including public health policies, high literacy rates and educational levels, the traditional diet and exercise levels, economic growth, and a stable political environment [24]. Since the mid to late 1990s, however, the pace of decline in mortality for adult Japanese men and, to a lesser extent, adult women (aged 15–59 years) has been slower than other nations [25]. If this trend continues, other nations are likely to achieve lower rates of adult mortality than Japan. Effective coverage of hypertension and hypercholesterolemia is also much lower in Japan than in other high-income countries [26, 27]. These situations may require not only changes of the Japanese health care system, but also a concerted effort to improve health literacy.

This study has several limitations. Health literacy in Japan was found to be lower than in Europe, but methodological issues must be addressed. The HLS-EU Survey involves a face-to-face interview, and this study used a Web-based self-administered questionnaire. Face-to-face interviews are more likely than any other mode to be affected by social desirability bias, because of the presence of an interviewer [28]. Anonymous Web surveys are likely to be least affected by this bias. If an interview survey had been used in Japan, the difference in health literacy might be smaller, but social desirability bias would certainly not account for all the difference.

It is possible that there was some sample selection bias. Participants in Japan may have been skewed toward a high level of Internet literacy because of the use of a Web-based survey. This also means that people who have problems with literacy or Internet use are included in the European sample, but not in the Japanese sample. Recruitment of respondents was based on self-selection from a group of individuals who had previously expressed a desire to participate in research projects. The responses were limited to approximately the first 1000 people, and may therefore only include those who are most active on the Internet (e.g., frequently checking e-mail). This implies that the differences between Japan and Europe might be even larger than shown in this survey.

Participants had a slightly higher education level and a larger municipality size; these factors might positively influence health status or health literacy. We therefore compared the health status of the sample with that of the largest national survey by the Japanese Ministry of Health, Labour and Welfare [17]. The combined proportions of “good” and “fairly good” in our study was 43.0 % in men and 50.8 % in women; in the ministry survey, about 39 % of men and 37 % of women responded similarly. In both the national survey and our own study, women had a higher level of health literacy than men. Although self-rated health decreased by age in the ministry survey, we observed little change by age in our study, and health literacy increased by age. The older participants in this Web-based survey might generally be more active than others, because they are fully utilizing new technology and participating in the survey. As a whole, the participants included in this survey may therefore be healthier than the average, in which case it would be unlikely for them to have lower health literacy than the average Japanese citizen. This suggests that the differences between Japan and Europe might be even greater than shown.

## Conclusion

This study translated a comprehensive health literacy questionnaire for which a Japanese version was not previously available, and confirmed the reliability and validity of this survey by conducting a Web-based cross-sectional nationwide survey. Comparative results of this questionnaire suggest that Japanese health literacy is lower than that of Europeans. This discrepancy may be partly caused by the inefficiency of the Japanese primary health care system. It is also difficult to access reliable and understandable health information in Japan as there is no comprehensive national online platform. Japanese respondents found it more difficult to judge and apply health information, which suggests that there may be difficulties in decision-making in health in Japan. The possible causes of low health literacy require further investigation.

## Additional file

Additional file 1:
**HLS-EU-Q47 Japanese version.**

## Abbreviations

HLS-EU: European Health Literacy Survey
GEN-HL: General health literacy index
HC-HL: Health care health literacy index
DP-HL: Disease prevention health literacy index
HP-HL: Health promotion health literacy index
CCHL: Communicative and critical health literacy
J-eHEALS: Japanese version of eHealth Literacy Scale
SRH: Self-rated health
MHI-5: Five-Item Mental Health Inventory
