# Supplementary material for: Comprehensive health literacy in Japan is lower than in Europe: a validated Japanese-language assessment of health literacy
Source: BMC Public Health. 2015 May 23;15:505. doi: 10.1186/s12889-015-1835-x (PMC4491868; doi:10.1186/s12889-015-1835-x)
Supplement: Additional file 1: — HLS-EU-Q47 Japanese version. [file 12889_2015_1835_MOESM1_ESM.docx]

HLS-EU-Q47 Japanese version (日本語版)

| 以下のそれぞれが、あなたにとって簡単か難しいかについてお聞きします。それぞれ「とても簡単」から「とても難しい」までで、最もあてはまるものに○を付けてください（それぞれひとつずつ）。 | | とても簡単 | やや 簡単 | やや 難しい | とても難しい | わからない/あてはまらない |
| --- | --- | --- | --- | --- | --- | --- |
| (1) | 気になる病気の症状に関する情報を見つけるのは | 1 | 2 | 3 | 4 | 5 |
| (2) | 気になる病気の治療に関する情報を見つけるのは | 1 | 2 | 3 | 4 | 5 |
| (3) | 急病時の対処方法を知るのは | 1 | 2 | 3 | 4 | 5 |
| (4) | 病気になった時、専門家（医師、薬剤師、心理士など）に相談できるところを見つけるのは | 1 | 2 | 3 | 4 | 5 |
| (5) | 医師から言われたことを理解するのは | 1 | 2 | 3 | 4 | 5 |
| (6) | 薬についている説明書を理解するのは | 1 | 2 | 3 | 4 | 5 |
| (7) | 急病時に対処方法を理解するのは | 1 | 2 | 3 | 4 | 5 |
| (8) | 処方された薬の服用方法について、医師や薬剤師の指示を理解するのは | 1 | 2 | 3 | 4 | 5 |
| (9) | 医師から得た情報がどのように自分に当てはまるかを判断するのは | 1 | 2 | 3 | 4 | 5 |
| (10) | 治療法が複数ある時、それぞれの長所と短所を判断するのは | 1 | 2 | 3 | 4 | 5 |
| (11) | 別の医師からセカンド・オピニオン(主治医以外の医師の意見)を得る必要があるかどうかを判断するのは | 1 | 2 | 3 | 4 | 5 |
| (12) | メディア（テレビ、インターネット、その他のメディア）から得た病気に関する情報が信頼できるかどうかを判断するのは | 1 | 2 | 3 | 4 | 5 |
| (13) | 自分の病気に関する意思決定をする際に、医師から得た情報を用いるのは | 1 | 2 | 3 | 4 | 5 |
| (14) | 薬の服用に関する指示に従うのは | 1 | 2 | 3 | 4 | 5 |
| (15) | 緊急時に救急車を呼ぶのは | 1 | 2 | 3 | 4 | 5 |
| (16) | 医師や薬剤師の指示に従うのは | 1 | 2 | 3 | 4 | 5 |
| (17) | 喫煙、運動不足、お酒の飲み過ぎなど不健康な生活習慣を改善する方法に関する情報を見つけるのは | 1 | 2 | 3 | 4 | 5 |
| (18) | ストレスや抑うつなどの心の健康問題への対処方法に関する情報を見つけるのは | 1 | 2 | 3 | 4 | 5 |
| (19) | 受けなくてはならない予防接種や検診（乳房検査、血糖検査、血圧）に関する情報を見つけるのは | 1 | 2 | 3 | 4 | 5 |
| (20) | 太りすぎ、高血圧、高コレステロールなどの予防法や対処法に関する情報を見つけるのは | 1 | 2 | 3 | 4 | 5 |
| (21) | 喫煙、運動不足、お酒の飲み過ぎなどの生活習慣が健康に悪いと理解するのは | 1 | 2 | 3 | 4 | 5 |
| (22) | 予防接種が必要な理由を理解するのは | 1 | 2 | 3 | 4 | 5 |
| (23) | 検診（乳房検査、血糖検査、血圧）が必要な理由を理解するのは | 1 | 2 | 3 | 4 | 5 |
| (24) | 喫煙、運動不足、お酒の飲み過ぎなどは健康に悪いといわれているが、その信頼性を判断するのは | 1 | 2 | 3 | 4 | 5 |
| (25) | 検査のために、いつ受診すべきかを判断するのは | 1 | 2 | 3 | 4 | 5 |
| (26) | どの予防接種が必要かを判断するのは | 1 | 2 | 3 | 4 | 5 |
| (27) | 必要な検診（乳房検査、血糖検査、血圧）の種類を判断するのは | 1 | 2 | 3 | 4 | 5 |
| (28) | メディア（テレビ、インターネット、その他のメディア）から得た健康リスク（危険性）の情報が信頼できるかどうかを判断するのは | 1 | 2 | 3 | 4 | 5 |
| (29) | インフルエンザの予防接種を受けるべきかどうかを決めるのは | 1 | 2 | 3 | 4 | 5 |
| (30) | 家族や友人のアドバイスをもとに、病気から身を守る方法を決めるのは | 1 | 2 | 3 | 4 | 5 |
| (31) | メディア（新聞、ちらし、インターネット、その他のメディア）から得た情報をもとに、病気から身を守る方法を決めるのは | 1 | 2 | 3 | 4 | 5 |
| (32) | 運動、健康食品、栄養などの健康的な活動に関する情報を見つけるのは | 1 | 2 | 3 | 4 | 5 |
| (33) | 心を豊かにする活動（瞑想[座禅・ヨガ]、運動、ウォーキング、ピラティスなど）について知るのは | 1 | 2 | 3 | 4 | 5 |
| (34) | より健康的な近隣環境にする方法（騒音や汚染を減らす、緑地やレジャー施設をつくるなど）に関する情報を見つけるのは | 1 | 2 | 3 | 4 | 5 |
| (35) | 健康に影響を与える可能性のある政策の変化（法律制定、新しい検診、政権交代、医療改革など）について知るのは | 1 | 2 | 3 | 4 | 5 |
| (36) | 職場の健康増進のための取り組みについて知るのは | 1 | 2 | 3 | 4 | 5 |
| (37) | 健康に関する家族や友人のアドバイスを理解するのは | 1 | 2 | 3 | 4 | 5 |
| (38) | 食品パッケージに書かれている情報を理解するのは | 1 | 2 | 3 | 4 | 5 |
| (39) | 健康になるためのメディア（インターネット、新聞、雑誌）情報を理解するのは | 1 | 2 | 3 | 4 | 5 |
| (40) | 心の健康を維持する方法に関する情報を理解するのは | 1 | 2 | 3 | 4 | 5 |
| (41) | 住んでいる場所（地域、近隣）がどのように健康と充実感に影響を与えているかを判断するのは | 1 | 2 | 3 | 4 | 5 |
| (42) | 住宅環境が健康維持にどのように役立つかを判断するのは | 1 | 2 | 3 | 4 | 5 |
| (43) | どの生活習慣（飲酒、食生活、運動など）が自分の健康に関係しているかを判断するのは | 1 | 2 | 3 | 4 | 5 |
| (44) | 健康改善のための意思決定をするのは | 1 | 2 | 3 | 4 | 5 |
| (45) | 参加したいときに、スポーツクラブや運動の教室に参加するのは | 1 | 2 | 3 | 4 | 5 |
| (46) | 健康と充実感に影響を与えている生活環境（飲酒、食生活、運動など）を変えるのは | 1 | 2 | 3 | 4 | 5 |
| (47) | 健康と充実感を向上させる地域活動に参加するのは | 1 | 2 | 3 | 4 | 5 |

(4)(12)(19)(23)(27)(28)(31)(33)(34)(35)(39)(41)(43)(46)でのかっこ内の例は、Table2では省略した。

作成代表者　中山和弘（聖路加国際大学看護学部）nakayama@slcn.ac.jp

オリジナルバージョン HLS-EU Consortium (2012): European Health Literacy Survey Questionnaire（HLS-EU-Q47）
